# Genome-wide association analysis of idiopathic epilepsy in the Belgian shepherd

**DOI:** 10.1186/s40575-020-00091-x

**Published:** 2020-09-10

**Authors:** J. M. Belanger, T. R. Famula, L. C. Gershony, M. K. Palij, A. M. Oberbauer

**Affiliations:** Department of Animal Science, University of California, One Shields Ave, Davis, CA 95616 USA

**Keywords:** Idiopathic epilepsy, ADAM23, Dog, Seizures, Epilepsy, GWAS

## Abstract

**Background:**

Idiopathic epilepsy (IE) is a common neurological disorder in the domestic dog, and is defined as repeated seizure activity having no identifiable underlying cause. Some breeds, such as the Belgian shepherd dog, have a greater prevalence of the disorder. Previous studies in this and other breeds have identified *ADAM23* as a gene that confers risk of IE, although additional loci are known to exist. The present study sought to identify additional loci that influence IE in the Belgian shepherd dog.

**Results:**

Genome-wide association studies (GWAS) revealed a significant association between IE and CFA 14 (*p* < 1.03 E^− 08^) and a suggestive association on CFA 37 (*p* < 2.91 E^− 06^) in a region in linkage disequilibrium with *ADAM23*. Logistic regression identified a 2-loci model that demonstrated interaction between the two chromosomal regions that when combined predicted IE risk with high sensitivity.

**Conclusions:**

Two interacting loci, one each on CFAs 14 and 37, predictive of IE in the Belgian shepherd were identified. The loci are adjacent to potential candidate genes associated with neurological function. Further exploration of the region is warranted to identify causal variants underlying the association. Additionally, although the two loci were very good at predicting IE, they failed to capture all the risk, indicating additional loci or incomplete penetrance are also likely contributing to IE expression in the Belgian shepherd dog.

## Lay abstract

Many dog breeds suffer from repeated seizure disorders. Idiopathic epilepsy, known to be inherited, is typically characterized as adult onset seizure activity with no identifiable underlying cause. Genetic association studies have been undertaken to reveal the causal DNA risk variants responsible for IE, although few significant genomic regions of association have been discovered. *ADAM23*, on canine chromosome 37 (CFA 37) has been identified as an IE gene common to many breeds. The present study investigated IE in the Belgian shepherd dog and found two regions of association, one on CFA 14 and a second suggestive region on CFA 37 in the vicinity of *ADAM23*. Using the statistical tool of logistic regression, the two regions were found to interact, with certain genotypes within the regions being associated with increased risk of IE in this breed.

## Background

Idiopathic epilepsy, or repeated seizure activity with no clear etiology, has been observed across mixed and purebred dogs [1, 2], although some breeds, such as Belgian shepherd, Irish Wolfhound, Labrador Retriever, Border Terrier, Petit Basset Griffon Vendén, Finnish Spitz, Italian Spinone, and German shepherd [3, 4] exhibit a higher prevalence. Despite epilepsy being the most common neurological condition in dogs [5], and IE likely having the presence of genetic risk factors, there have been great challenges in identifying the genetic underpinnings of the disorder. It is well understood that in most cases IE is polygenic with particular genotypes contributing to risk [4, 6–9]. It is also speculated that each affected breed may have its own breed-specific genetic susceptibility profile that interacts with other risk loci that are common across breeds. Although there may be instances of specific seizure disorders presenting as a monogenic condition, at this time only in the Lagotto Romagnolo breed has a single gene (*LGI2*) influencing IE been identified [10] and a deletion in the *DIRAS* gene is associated with generalized juvenile myoclonic epilepsy in Rhodesian ridgeback dogs [11]. In contrast, many single genes have been identified as responsible for other forms of epilepsy such as the progressive myoclonic epilepsies in which a metabolic disturbance underlies the seizure episodes: *NHLRC1/EPM2B* for Lafora disease and *CLN8, CLN5, CTSD, TPP1, PPT1, ARSG, CLN6, ATP13A2* for neuronal ceroid lipofuscinosis [2]. The prevalence of IE in the Belgian shepherd is a recognized health concern in the breed and breeders have sought means to reduce the incidence [12]. Although the prevalence appears to have decreased with time [13], owners and breeders would like more specific breeding tools, including genetic testing, to further decrease it. Approaches to identify risk variants leading to IE have relied upon candidate gene analysis and genome-wide association studies (GWAS) [7, 14–17]. Within the Belgian shepherd, a risk factor for IE was identified in *ADAM23* but additional suggestive loci were noted [16]. The present study sought to identify additional IE risk loci in Belgian shepherd dogs using well-phenotyped cases, aged controls, and the high-density Illumina single nucleotide polymorphism (SNP) array to increase the likelihood of loci discovery.

## Results

**Fig. 1:**
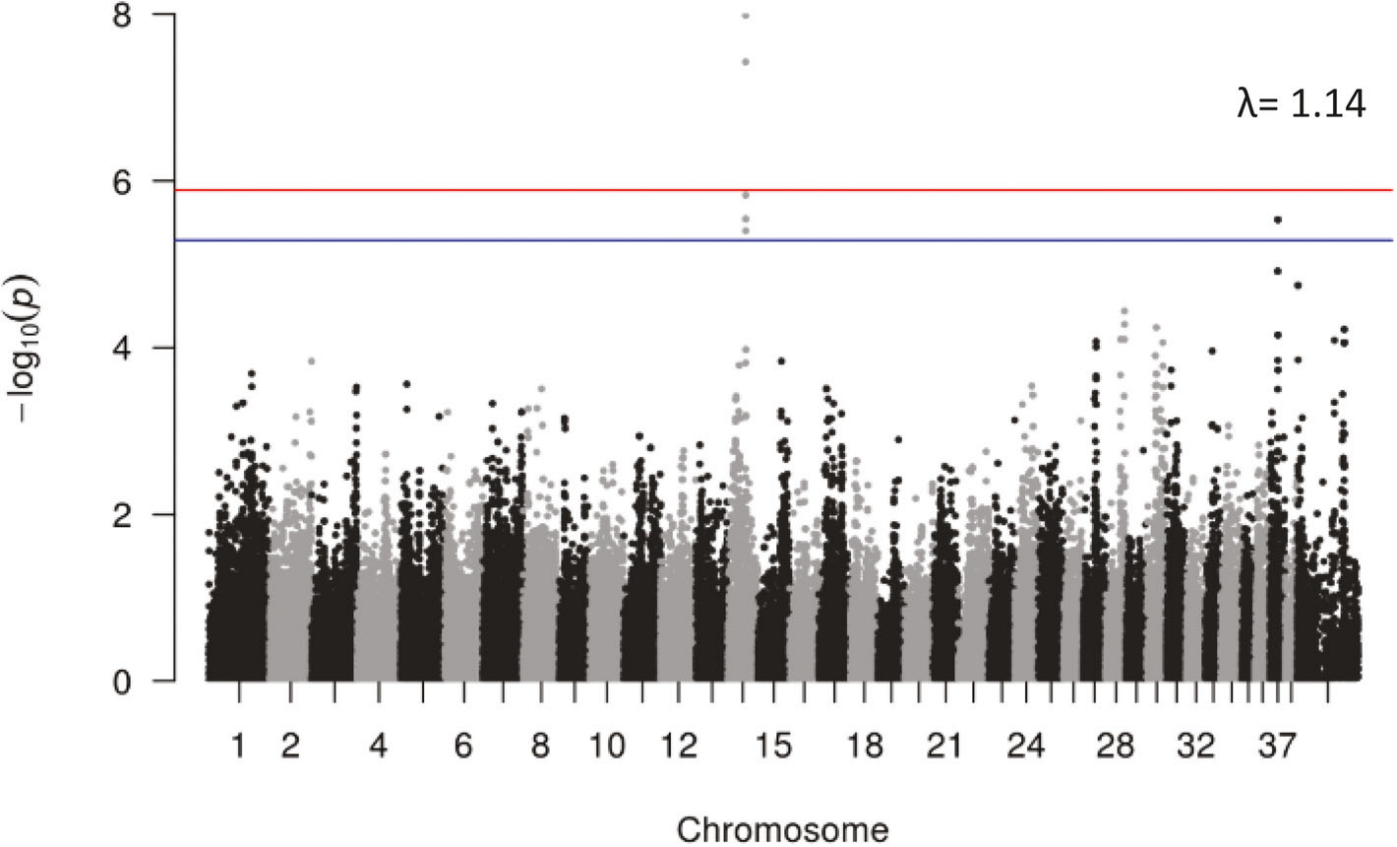
Manhattan plot of association testing for idiopathic epilepsy for the entire study cohort using GEMMA’s univariate linear mixed model approach to account for population substructure. The red line indicates the genome-wide significance threshold defined as *p* < 0.05/NES and the blue line indicates genome-wide suggestive significance threshold defined as *p* < 0.20/NES where NES is the number of effective SNPs

Following SNP genotyping quality control, related individuals were removed leaving 20 IE cases and 45 controls that met the criteria outlined in the methods. Association testing was then performed using GEMMA’s linear mixed model approach. A distinct peak of association with IE was noted on CFA 14 with three SNPs reaching genome-wide significance (BICF2P1043850, BICF2S23749130, and TIGRP2P191770_rs9025240) and four reaching suggestive significance (BICF2S23230472, BICF2P437468, BICF2S23211419, and BICF2S23539344). Two SNPs (BICF2P271491 and TIGRP2P419463_rs8724220) reached suggestive significance on CFA 37 (Fig. 1). The SNPs on CFA 37 align with a region adjacent to, and in strong linkage disequilibrium (D’ = 1, *r*^2^ = 0.68) with the *ADAM23* locus, the locus previously described as a modest IE risk locus in the Belgian shepherd [16].

The two Belgian shepherd varieties were then analyzed separately as it has been suggested that lineages may possess different risk variants [18]. In neither the Belgian sheepdog nor the Belgian Tervuren variety did any SNP reach significance, likely reflecting small subset sample numbers (data not shown).

The odds ratios (OR) for the significant (CFA 14) and suggestive SNPs (CFA 37) were calculated on the entire study cohort (Tables 1 and 2); OR were also calculated on the remaining samples after the relatedness cutoff was performed and similar results were obtained (data not shown). Strong linkage disequilibrium was seen between the three significant SNPs on CFA14 (D’ = 1 and *r*^2^ = 1 between BICF2S23749130 and TIGRP2P191770_RS9025240, and D’ = 1 and *r*^2^ = 0.96 between these two SNPs and BICF2P1043850) with these segregating together despite spanning nearly 80,000 bp with intervening SNPs that did not. A similar situation was present for the two suggestive SNPs on CFA 37 in that they also appeared to segregate together even though they were more than 70,000 bp apart: D’ = 1 and *r*^2^ = 1 for BICF2P271491 and TIGRP2P419463_rs8724220. Dogs that were homozygous for alleles on CFA 14 (e.g., BICF2P1043850-AA, BICF2S23749130-AA, and TIGRP2P191770_rs9025240-AA) were at reduced risk of IE versus those homozygous (GG) or carriers (AG) of the alternate allele at these three SNPs (Table 3). For the CFA 37 SNPs, increased risk was observed in dogs that were homozygous GG at both BICF2P271491 and TIGRP2P419463_rs8724220. The presence of even one A allele at the suggestive SNPs was associated with significantly reduced risk of IE.

**Table 1:** Odds Ratios (OR) and 95% confidence intervals (CI) for the CFA14 SNPs associated with idiopathic epilepsy that reached genome wide significance. The CanFam3.1 reference allele is indicated in bold, and the allele associated with elevated risk for the Belgian shepherd is indicated with an asterisk

SNP name	Location (bp)	Allele	Controls (2n = 124)	Cases (2n = 70)	OR (95% CI)	*p* value
**BICF2P1043850**	36,043,569	A	108	41	0.21 (0.10–0.43)	0.000010
		G*	16	29		
**BICF2S23749130**	36,098,932	A	109	41	0.19 (0.09–0.40)	0.000005
		G*	15	29		
**TIGRP2P191770_rs9025240**	36,123,553	A	109	41	0.19 (0.09–0.40)	0.000005
		C*	15	29

**Table 2:** Odds Ratios (OR) and 95% confidence intervals (CI) for the suggestive SNPs associated with idiopathic epilepsy on CFA 37. The CanFam3.1 reference allele is indicated in bold and the allele associated with elevated risk for the Belgian shepherd is indicated with an asterisk

SNP name	Location (bp)	Allele	Controls (2n = 124)	Cases (2n = 70)	OR (95% CI)	*p* value
**BICF2P271491**	15,527,401	A	49	8	0.20 (0.09–0.45)	0.000031
		G*	75	62		
**TIGRP2P419463_rs8724220**	15,597,532	A	49	8	0.20 (0.09–0.45)	0.000031
		G*	75	62

**Table 3:** Odds Ratios (OR) and 95% confidence intervals (CI) for genotypes of the 3-SNP CFA 14 haplotype and the 2-SNP CFA 37 haplotype associated with idiopathic epilepsy. Not applicable (NA) reflects the inability to calculate the OR. The genotype associated with elevated risk for Belgian shepherds is indicated with an asterisk

**Genotype for the significant CFA 14 SNPs BICF2P1043850/BICF2S23749130/TIGRP2P191770_rs9025240**	**Controls (n = 62)**	**Cases (n = 35)**	**OR (95% CI)**	***p*** **value**
AA/AA/AA	46	9	0.12 (0.05–0.31)	0.000005
AG/AG/AC*	16	23	5.51 (2.24–13.56)	0.000201
GG/GG/CC	0	3	NA	0.044391
AA/AA/AA vs GG/GG/CC	46	9	NA	0.007130
	0	3		
AA/AA/AA vs AG/AG/AC*	46	9	0.14 (0.05–0.35)	0.000024
	16	23		
AG/AG/AC vs GG/GG/CC	16	23	NA	0.275261
	0	3		
**Genotypes for the suggestive CFA 37 SNPs BICF2P271491/TIGRP2P419463_rs8724220**	**Controls (n = 62)**	**Cases (n = 35)**	**OR (95% CI)**	***p*** **value**
AA/AA	12	1	0.12 (0.02–0.99)	0.028124
AG/AG	25	6	0.31 (0.11–0.84)	0.023494
GG/GG*	25	28	5.92 (2.24–15.62)	0.000249
AA/AA vs GG/GG*	12	1	0.07 (0.01–0.61)	0.004025
	25	28		
AA/AA vs AG/AG	12	1	0.35 (0.04–3.22)	0.419318
	25	6		
AG/AG vs GG/GG*	25	6	0.21 (0.08–0.61)	0.002890
	25	28		

**Fig. 2:**
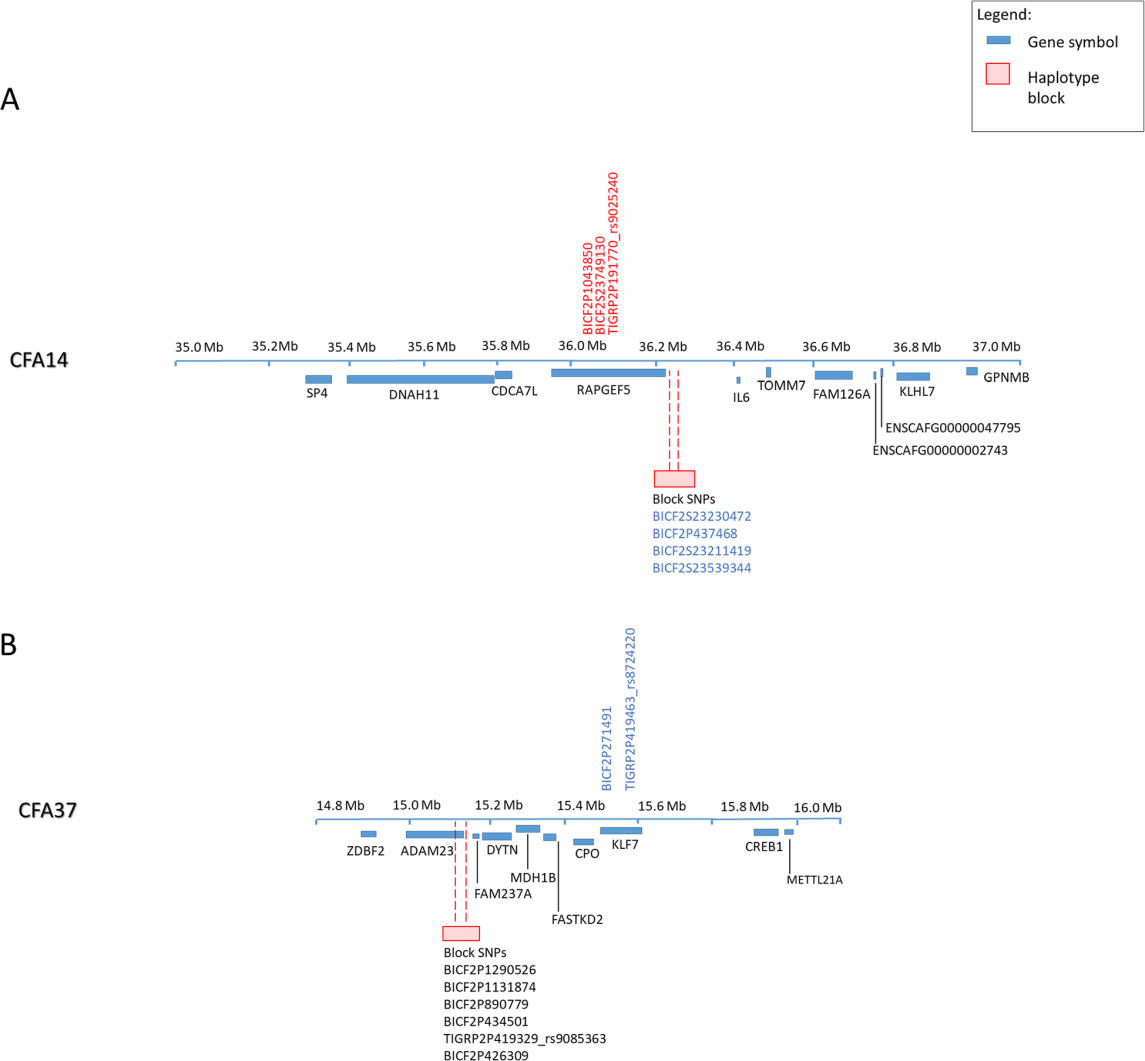
Diagram of the locations of the GWAS SNPs and haplotype blocks on (**a**) CFA14 and (**b**) CFA37. Genome-wide significant SNPs are indicated in red, SNPs reaching suggestive significance are indicated in blue

The three significant SNPs on CFA 14 identified from the GWAs fell within an eight-SNP haplotype block, and while the individual SNPs remained significant after 25,000 permutations, the block as a whole failed to reach significance. A single significant haplotype block (*p* < 0.00004) was identified on CFA 14 and one on CFA 37, within the group of dogs that remained after the relatedness cutoff was applied (20 IE cases and 45 healthy controls) (Table 4 and Fig. 2). The block on CFA 14 encompassed the four suggestive SNPs identified in the GWAS analysis and spanned a 60,314 bp region (CFA 14: 36227069–36,287,383 bp). The significant haplotype block on CFA 37 included six SNPs spanning 72,024 bp (CFA 37: 15,093,174 - 15,165,198), including the *ADAM23* gene and published associated SNPs (Supplemental Table 1), though none of those SNPs reached suggestive significance on their own. For the haplotype block on CFA 14, 56.8% of control dogs were homozygous with a CTCT genotype as compared to only 5% of the IE cases.

**Table 4:** Odds Ratio for the significant haplotype blocks on CFA 14 and 37. The CanFam3.1 reference allele is indicated in bold and haplotypes associated with significantly elevated risk for idiopathic epilepsy in Belgian shepherds are indicated with an asterisk

**CFA 14 Haplotype Block**^a^	**Haplotype**	**Controls (2n = 88)**	**Cases (2n = 38)**	**OR (95% CI)**	***p*** **value**
BICF2S23230472 BICF2P437468 BICF2S23211419 BICF2S23539344	**C**T**CT**	67	14	0.18 (0.08–0.42)	0.00004
	vs. **C**T**C**G, A**C**TG	21	24		
	**C**T**C**G	14	9	1.64 (0.64–4.20)	0.321651
	vs. **C**T**CT,** A**C**TG	74	29		
	A**C**TG*	7	15	7.55 (2.75–20.71)	0.000054
	vs. **C**T**CT, C**T**C**G	81	23		
**CFA 37 Haplotype Block**	**Haplotype**	**Controls (2n = 90)**	**Cases (2n = 40)**	**OR (95% CI)**	***p*** **value**
BICF2P1290526 BICF2P1131874 BICF2P890779 BICF2P434501 TIGRP2P419329_rs9085363 BICF2P426309	**TAGCTC**	43	4	0.12 (0.04–0.37)	0.000057
	vs. **T**GA**CTC,** CGAT**TC**, CGATGT	47	36		
	**T**GA**CTC**	24	16	1.83 (0.84–4.03)	0.151538
	vs **TAGCTC**, CGAT**TC**, CGATGT	66	24		
	CGATTC*	10	13	3.85 (1.52–9.79)	0.00548
	vs. **TAGCTC**, **T**GA**CTC**, CGATGT	80	27		
	CGATGT	13	7	1.26 (0.46–3.43)	0.7927
	vs. **TAGCTC**, **T**GA**CTC,** CGAT**TC**	77	33		

^a^ genotype for BICF2S23230472 was unable to be determined for one case and one control dog

**Fig. 3:**
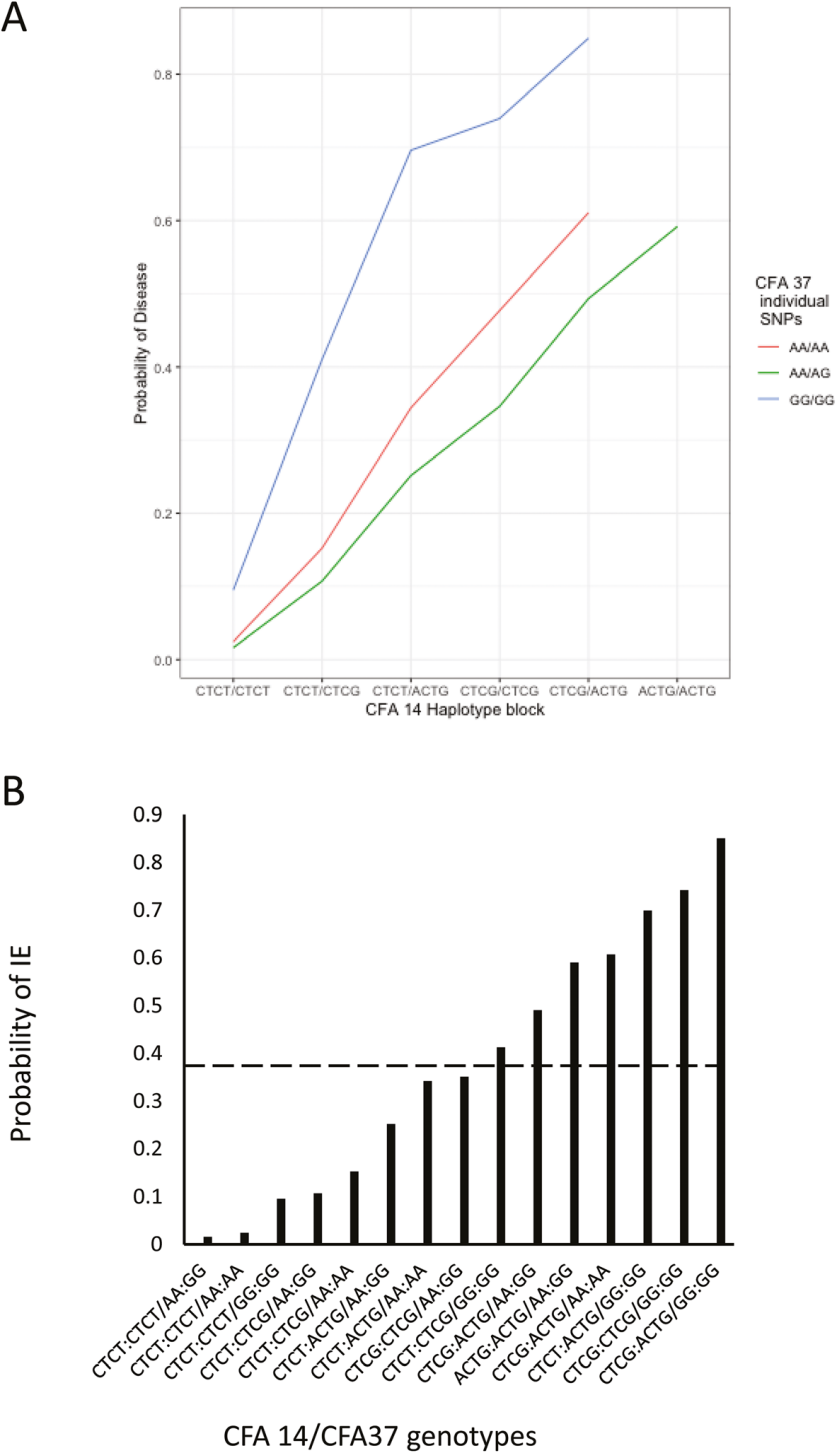
Interactions of the CFA 14 haplotype block genotypes and the CFA 37 suggestive SNP genotypes on the probability of idiopathic epilepsy (IE) in the Belgian Shepherds (**a**) and as a function of specific genotypic interactions (**b**). The dashed line in B indicates the computed threshold (0.382) and probabilities in excess of the line are at increased risk of IE

With the hypothesis that IE is multigenic, logistic regression was applied to determine if these four regions, two on each of CFA 14 and CFA 37, interacted and whether the genetic association with IE would be strengthened if more than one region was considered. That is, the two regions on CFA14 (the individual SNPs that were genome-wide significant BICF2P1043850, BICF2S23749130, and TIGRP2P191770_rs9025240 and the significant haplotype block of BICF2S23230472, BICF2P437468, BICF2S23211419, and BICF2S23539344) and the two regions on CFA 37 (the suggestive SNPs:BICF2P271491 and TIGRP2P419463_rs8724220 and the significant haplotype block of BICF2P1290526, BICF2P1131874, BICF2P890779, BICF2P434501, TIGRP2P419329_rs9085363, and BICF2P426309) were analyzed to determine which loci might be interacting. Specifically, using the known genotypes and associated phenotypes, the probability of IE associated with every possible genotypic combination was calculated by logistic regression. The model was designed to address whether a multi-SNP model improved the ability to predict IE. The analysis of 2-loci, 3-loci and 4-loci models included the 20 IE cases and 45 healthy controls that remained after the relatedness cutoff was applied. Balancing the looic values, significant SNPs, as well as specificity and sensitivity of the models, the best and most parsimonious fit was observed for the 2-loci model that included the CFA 14 haplotype block and the two suggestive SNPs on CFA 37 considered as a 2-SNP haplotype (Fig. 3a). The estimated threshold of that model was 0.382, meaning a predicted probability greater than the threshold predicts that individual would be observed as an affected dog (Fig. 3b). This threshold, using the present data, resulted in a specificity of 0.795, meaning that a considerable fraction of false positives for disease would be expected. However, this threshold also resulted in a sensitivity of 0.947, suggesting that this model would be very good at predictions. Note further, that based upon a 0.30 prevalence of IE in this study cohort, the positive predictive value was 0.667 and the negative predictive value was 0.972. The positive predictive value indicates that if the dog had the risk alleles then ~ 67% of those dogs would be expected to express IE and the negative predictive value indicates that ~ 97% of dogs without the risk alleles would be free from IE. The confidence in those predictive values would increase if the prevalence of IE in the breed at large is below the 0.30 value of this study cohort.

Dogs that were homozygous CTCT for the 4-SNP CFA 14 haplotype block were associated with the lowest risk range of IE in this study cohort. Being heterozygous or homozygous for the other alleles increased the risk of IE. For the suggestive individual SNPs on CFA 37, being homozygous (AA:AA) or heterozygous (AA:GG) reduced the risk of IE. The greatest risk of disease was observed for dogs that were heterozygous for the alleles in the CFA 14 haplotype block (CTCG:ACTG) in addition to being homozygous (GG:GG) at the two suggestive SNPs on CFA 37.

It has been proposed that, for some breeds, the genetic profile of IE dogs refractory to antiepileptic drugs (AED) may differ from the other IE dogs who are responsive to AED [19]. To address AED responsiveness as a potential genetic differentiator, we assessed a very small subset of dogs (*n* = 5 BT) that had full and detailed medical notes indicating that the IE was fully controlled and seizures had stopped entirely with administration of phenobarbital. Because of the small sample size and this effort not being the main overall thrust of the research, the results of this analysis are presented as supplementary material (Supplemental File 2).

## Discussion

In the present study, a GWAS using the Illumina 173 k SNP array identified genomic associations with IE on CFA 14 and CFA 37 in the Belgian shepherd dog. Although the sample size was limited, the cases and controls were robustly defined due to strict criteria applied for phenotypic classification. Previous studies have identified *ADAM23* on CFA 37 to be associated with IE in the Belgian shepherd as well as other breeds [6, 16]. The significant association of the haplotype block that includes *ADAM23* SNPs seen here in a different group of dogs further validated the previous finding and confirmed that the present study cohort was representative of the breed at large. Specifically, two SNPs in our significant haplotype block, BICF2P1131874 and BICF2P890779, were reported to be highly associated with IE in the Belgian shepherd breed [16, 20]. The association on CFA 14 has not been reported before.

Knowing that IE is multigenic, the interplay between the two associated chromosomal regions was assessed. When combined, two regions were most informative at predicting risk of IE in this study cohort: the CFA 14 haplotype block upstream of the Rap guanine nucleotide exchange factor (*RAPGEF5*) gene which lies on the minus strand and the two single SNPs that segregate together on CFA 37 within the Kruppel-like factor 7 (*KLF7*) gene. The region that included the previously identified *ADAM23* SNPs did not improve predictive power over that of the *KLF7* SNPs when paired with those on CFA 14.

The nearest gene to the CFA 14 block is the *RAPGEF5* gene that activates Rap proteins and plays a role during neurogenesis [21]. The downregulation of *RAPGEF5* has been associated with “fast ripples”, a term applied to the altered electrophysiology of the temporal lobe that characterizes the onset of focal seizures [22]. Genetic variants in *RAPGEF5* have been tentatively associated with human perception and facial recognition [23]. The haplotype block was located 17,855 bases upstream of *RAPGEF5* perhaps suggesting variants could play a role in modulating the promoter activity and influence susceptibility to seizures in the Belgian shepherd.

Kruppel-like factor 7 is important in neurological development, with broad expression throughout the brain during prenatal development [24], and essential for survival as shown in a knockout mouse model [25]. Beyond its developmental role, neural expression of *KLF7* persists throughout adulthood and is also proposed to play a role in neurological maintenance [26] making it an excellent candidate gene for IE. Prior sequencing of the *KLF7* exons in Belgian shepherds failed to demonstrate variation between IE and control dogs whereas sequencing of *ADAM23* uncovered a strongly associated variant in exon 12 [16]; the authors concluded that this variant was likely a linked polymorphism and not the causal variant. The data of the present study supports this concept and suggests that the actual causal variant lies somewhere within this extensive CFA 37 region (Supplemental File 1 Supplemental Table 1). Whole genome or additional targeted sequencing will be necessary to more fully characterize the association and identify the functional variant.

In the published literature for canine epilepsy, there are many identified genes that regulate myoclonic epilepsy [2], and focal seizures associated with fear or aggression have also been considered hereditary in certain dog breeds, with suspected risk variants uncovered [27, 28]. Only for the Lagotto Romagnolo [10] and the Rhodesian ridgeback [11] has a single gene influencing IE been identified. Other attempts to identify association have been less successful. As noted previously, *ADAM23* was uncovered by GWAS and a significant haplotype association between IE and a region on CFA 4 was identified in Irish Wolfhounds [17]. The present study demonstrated a genome-wide significant association on CFA 14 that interacts with a distinct CFA 37 region; when risk alleles were present on both chromosomes the probability of IE increased. Candidate genes are present in both associated regions and deep sequencing of the regions may uncover causal variants.

It is notable that the model judged as being the best fit had high sensitivity in predicting dogs with IE but the specificity was less robust, meaning that more false positives would occur. Specifically, based upon the IE prevalence in the study cohort, it was estimated that ~ 33% of dogs that carry the risk alleles would not exhibit IE. In contrast, false negatives were ~ 2% suggesting that absence of the risk alleles was highly associated with being free of IE. With complex, multigenic disorders, many factors influence the manifestation of disease and the presence of risk alleles may be predisposing but not sufficient. Contributing factors may be environmental exposures or additional small effect loci that have not yet been identified. Epilepsy has often been viewed as requiring multiple genes and or environmental triggers in order to surpass a threshold to evoke clinical signs. The proportion of false positives predicted by the present study reinforces that view. Implementing knowledge of the presence or absence of risk alleles demands caution. In breeding decisions, where one is trying to reduce the overall incidence of IE in the breed, it may be prudent to remove any dog that might be affected, even those mistakenly identified as such; however, it is vital to avoid overly restricting the population and removing individuals who have attributes that could be important to the breed as a whole. Furthermore, knowing that additional, and as of yet unknown, factors are at play in the expression of IE in this breed, and given the lower specificity and its concomitant potential to reduce population size due to false positives, application of these findings in any type of selection scheme is premature.

Despite the samples being well characterized and the finding of significant association and demonstrated interaction between the two chromosomes, any single study on a small cohort should be interpreted with caution. However, this study did replicate previous work that has shown an association between CFA 37 and IE in this breed. Nonetheless, additional studies with larger cohorts are warranted to validate the observed association on both chromosomes, more accurately characterize the sensitivity and specificity estimations of interaction, and identify a causal variant. Furthermore, multigenic traits often represent complex interactions between genes and environmental inputs, which may obscure important genetic contributions. An additional difficulty of identifying genetic variants underlying IE in the Belgian shepherd, beyond a multigenic nature, is the possibility that subgroups within a single breed could have different independent mutations that result in similar generalized seizure phenotypes. Thus, our findings are preliminary awaiting further validation in future studies, yet provide insight toward uncovering the genetics underlying IE in the dog.

## Conclusions

Two interacting loci, one each on CFA 14 and CFA 37, which were predictive of IE in the Belgian shepherd were identified. The loci are adjacent to potential candidate genes associated with neurological function, and therefore provide guidance to exploring the genome more fully for causal variants. The study findings also underscored the multigenic nature of the disease. The two loci logistic model was very good at predicting IE in this study cohort. However, even in the presence of the highest risk alleles for each of these two loci, only a portion of dogs would be predicted to express IE. This points to the existence of additional, as yet unidentified, contributors. Nevertheless, the findings reinforced the previous findings of a CFA 37 locus (*ADAM23*) involvement and suggested another interacting locus on CFA 14 associated with IE in the dog.

## Methods

### Study cohort

The study cohort consisted of 97 Belgian shepherds of the Sheepdog (BS, Groendael *n* = 30) and Tervuren (BT, *n* = 67) varieties. Dogs were classified as either IE cases or controls. Cases (*n* = 35 of which 15 were BS and 20 were BT) were defined as dogs that had repeated generalized seizures with an age of onset above 12 months of age (median age 36 months), which is more associated with inherited IE [9], and no other explanatory causes or underlying health conditions as detailed by owners through questionnaires, with characterization similar to previously employed strategies for Belgian shepherds [29]. Only two of the cases had an onset at over 6 years of age, one at 76 months and one at 96 months. Both had full neurological work ups including MRIs at university teaching hospitals and all physical causes for seizures were ruled out. The 8 year old case was excluded from the GWAS analyses for relatedness. Using the criteria for diagnosis proposed by the International Veterinary Epilepsy Task Force [30], cases were classified as Tier 1 based upon owner, and when available veterinarian, comments and descriptions. Controls (*n* = 62, of which 15 were BS and 47 were BT) were limited to dogs over the age of 10 years (median age 12.8 years) without any neurologic or immune-mediated disease. A small subset of BT dogs (*n* = 5) was identified by further narrowing down the IE phenotypic criteria to individuals for which the IE was fully controlled by phenobarbital alone (Supplemental File 2). Blood or buccal swab samples were obtained and the DNA extracted as previously described [31]. DNA aliquots were quantified using a Nanodrop® spectrophotometer and stored at − 20 °C until further analysis. The study followed all applicable guidelines for the institutional care and use of animals in accordance with the ethical standards of the University of California, Davis.

### Genome-wide association study

DNA samples were genotyped at GeneSeek (Lincoln, NE) using the Illumina CanineHD BeadChip (San Diego, CA) containing 173,662 single nucleotide polymorphisms (SNPs). Data filtering was used to remove individuals and SNPs with less than 95% call rates, SNPs with a minor allele frequency less than 5% as well as SNPs deviating from Hardy-Weinberg equilibrium among the controls (*p* < 0.0001). Prior to association testing, closely related individuals were excluded using a relatedness cutoff of 0.3 in PLINK [32]. Although samples were selected to be mostly unrelated, and the relatedness cutoff excludes closely related dogs, population structure is an issue for breeds with small effective population size; therefore GEMMA version 0.97 was used to account for genetic relatedness on the remaining samples. False positive associations were minimized without being overly stringent by using Bonferroni-corrected thresholds based on the number of effective SNPs (NES) determined by the Genetic Type I error calculator (GEC) after linkage disequilibrium pruning [33]. Thus, significant SNPs were defined as those with *p* < 0.05/NES, whereas suggestive SNPs were defined as *p* < 0.20/NES. Genomic inflation factors were calculated and Manhattan plots were plotted in R [34] using the package qqman [35]. To better define the associated regions of the GWAS on CFA 14 and CFA 37, Haploview version 4.2 was used to identify haplotype blocks on the chromosomes showing association and 25,000 permutations to were run to determine the significance (pperm < 0.05) of haplotype and single marker associations with IE [20, 36]. The D’ and *r*^*2*^ statistics to indicate linkage disequilibrium between SNPs was calculated by Haploview. The odds ratio (OR) based on the number of IE cases and healthy controls carrying a particular allele or genotype as compared to the number of cases and controls not carrying that particular allele or genotype and two-tailed Fisher’s exact *p*-values were calculated using a 2 × 2 contingency table in VassarStats (http://vassarstats.net/odds2x2.html). The ORs were also calculated for genotypic pairwise comparisons to determine which genotypes conferred increased risk of IE. Statistical significance was considered at *p* < 0.05. The dataset supporting the conclusions of this article is included within the article and genotypes are in Supplemental File 3 Supplemental Table 2.

The risk of disease was modeled across the unrelated dogs by logistic regression using the observed genotypes at loci identified as being significant. Disease probability was defined as *p*_*i*_ for the i-th dog identified across k loci genotypes and the logit of this probability as *θ*_*i*_ =  *log* [*p*_*i*_/(1 − *p*_*i*_)]. The logit as a function of the genotypes used this linear model:

1 $$ {\theta}_i={b}_0+{\sum}_{j=1}^k{b}_j{SNP}_j $$

where *b*_0_ was an unknown constant common to all dogs and *b*_*j*_ was the contribution of the j-th locus of the *k* loci being considered in the analysis. Estimation of the unknown genotypic effects (*b*_*j*_), and predictions of the risk of disease, were done with the Bayesian statistical package Stan [37] using the public domain language R [34]. A hierarchical Bayesian model with weakly informative prior distributions for the unknown effects helped stabilize the estimation process [38], which was necessary because the combination of rare genotypes with a low prevalence of disease increases the risk of empty subclasses.

Specifically, the intercept (*b*_0_) and genotypic effects (*b*_*j*_) were drawn from the prior density Cauchy (0,2.5) as recommended for non-hierarchical logistic regression [39]. The simulation process was conducted across 4 chains, where each chain was built on a draw of 25,000 total samples, a “burn-in” process of 5000 samples followed by thinning to every 25-th sample. Each chain generated 800 samples, and 3200 samples across the 4 chains. Convergence of the process was visualized through trace plots of all the unknown values and computation of the Gelman-Rubin statistic for convergence being below 1.05 [40]. Following fitting the various models based upon the multiple combinations of the presumptive important loci, the predictive capacity of the models were assessed using the leave-one-out cross-validation process, adapted for Bayesian analysis [41]. The leave-one-out information criterion (looic) statistic, is similar to that of the Akaike Information Criterion (AIC), where the model with the smallest value for the information criterion is considered the best, most parsimonious, fit although other model attributes must be considered such as loci significance, sensitivity and specificity. The implementation of looic measure of a model’s predictive accuracy was performed with the R package loo [41]. The value of the models in the prediction of disease was assessed through the receiver operating characteristic curve, fitted with the R package pROC [42]. The overall objective of these models was to determine the optimal discrimination threshold for assigning normal or affected status to dogs using the predictions of the best logistic regression model from each dog’s known SNP/loci genotypes.

## Supplementary information

**Additional file 1: Supplemental Table 1** Summary of current and published CFA 37 SNPs, and their locations, associated with IE in the Belgian shepherd dog.

**Additional file 2.** GWAS analyses of Belgian Tervuren whose IE was responsive to phenobarbital.

**Additional file 3: Supplemental Table 2** provides the genotypes for the associated regions on CFA 14 and CFA 37 for all dogs in the study cohort. The data was used in the predictive risk analysis between the four regions (two on CFA14 and two on CFA37). The genotypes are listed as CFA14 haplotype block, CFA14 single significant SNPs as a block, CFA37 Haplotype block and CFA37 single suggestive SNPs as a block.

## Data Availability

All genotype data analyzed during this study are included in this published article [and its supplementary information files].
