# Supplementary material for: Genome-wide association analysis of idiopathic epilepsy in the Belgian shepherd
Source: Canine Med Genet. 2020 Sep 10;7:12. doi: 10.1186/s40575-020-00091-x (PMC7491283; doi:10.1186/s40575-020-00091-x)
Supplement: Supplementary file 2 — Additional file 2. GWAS analyses of Belgian Tervuren whose IE was responsive to phenobarbital. [file 40575_2020_91_MOESM2_ESM.docx]

**Supplemental File 2:**

Phenobarbital responsive GWAS

Only BT controls (n = 47) were used in this comparison because the data for phenobarbital responsive dogs were all from BT. The GEMMA analysis done after the relatedness cutoff was applied revealed a strong association with CFA 8 and four SNPs reached genome-wide significance (Figure 1). The OR values are presented in Table 1; note that the 95% confidence intervals for the estimates were inordinately broad due to the limited number of dogs. Two significant haplotype blocks were identified: one spanning a large 115,743 bp region (BICF2P507188, BICF2S23424036, BICF2P1013837, BICF2S23011483, BICF2P1028102) that included the most significant SNP in the GWAS and another spanning a 78,165 bp region (BICF2P413589, BICF2P316979, BICF2S23434759, BICF2S23214357, BICF2P795913) that also contained a significant GWAS SNP.

The phenobarbital-responsive epilepsy GWAS was performed to achieve greater specificity by controlling for additional risk variables based on the premise that dogs equally responsive to the same medication might have similar underlying mechanisms for their disease. Thus, limiting cases based on that information could help ensure phenotypic consistency among cases. Our study uncovered significant SNPs and haplotypes on CFA 8 associated with IE among phenobarbital responsive dogs that were in gene poor regions at quite a distance from genes involved in neurological function or ion channel regulation. While this association is intriguing, the limited sample size, the rather high genomic inflation factor, and the allele frequencies of associated SNPs require extreme caution in interpretation.

Published studies of AED in dogs have focused primarily on AED refractoriness with attention centered on the ATP binding cassette subfamily B member 1 (*ABCB1* also known as *MDR1*) due to its role in modulating drug responsivity. A research group found a deletion in an intron in the *ABCB1* gene associated with refractoriness to AEDs in border collies [1]. Another group reported an association of an *ABCB1* variant (*ABCB1* c.6-180T>G) with AED resistance in border collies [2], whereas a more widespread association across multiple breeds was not found in follow up studies [3]. Although in the present study the sample size was extremely restricted and the subset consisted of dogs responsive to AED, no association between the *ABCB1* SNPs and the phenobarbital responsive dogs with IE was observed.

1. Alves L, Hülsmeyer V, Jaggy A, Fischer A, Leeb T, Drögemüller M. Polymorphisms in the ABCB1 gene in phenobarbital responsive and resistant idiopathic epileptic Border Collies. Journal of veterinary internal medicine. 2011;25(3):484-9.

2. Mizukami K, Yabuki A, Chang H-S, Uddin MM, Rahman MM, Kushida K, et al. High frequency of a single nucleotide substitution (c.-6-180T> G) of the canine MDR1/ABCB1 gene associated with phenobarbital-resistant idiopathic epilepsy in Border Collie dogs. Disease markers. 2013;35(6):669-72.

3. Gagliardo T, Gandini G, Gallucci A, Menchetti M, Bianchi E, Turba M, et al. ABCB1 c.-6-180T> G polymorphism and clinical risk factors in a multi-breed cohort of dogs with refractory idiopathic epilepsy. The Veterinary Journal. 2019;253:105378.

Supplemental File 3 Figure 1. Manhattan plot of association testing for idiopathic epilepsy for the phenobarbital responsive BT subset using GEMMA’s univariate linear mixed model approach to account for population substructure. The red line indicates the genome-wide significance threshold defined as p < 0.05/NES and the blue line indicates genome-wide suggestive significance threshold defined as p < 0.20/NES where NES is the number of effective SNPs


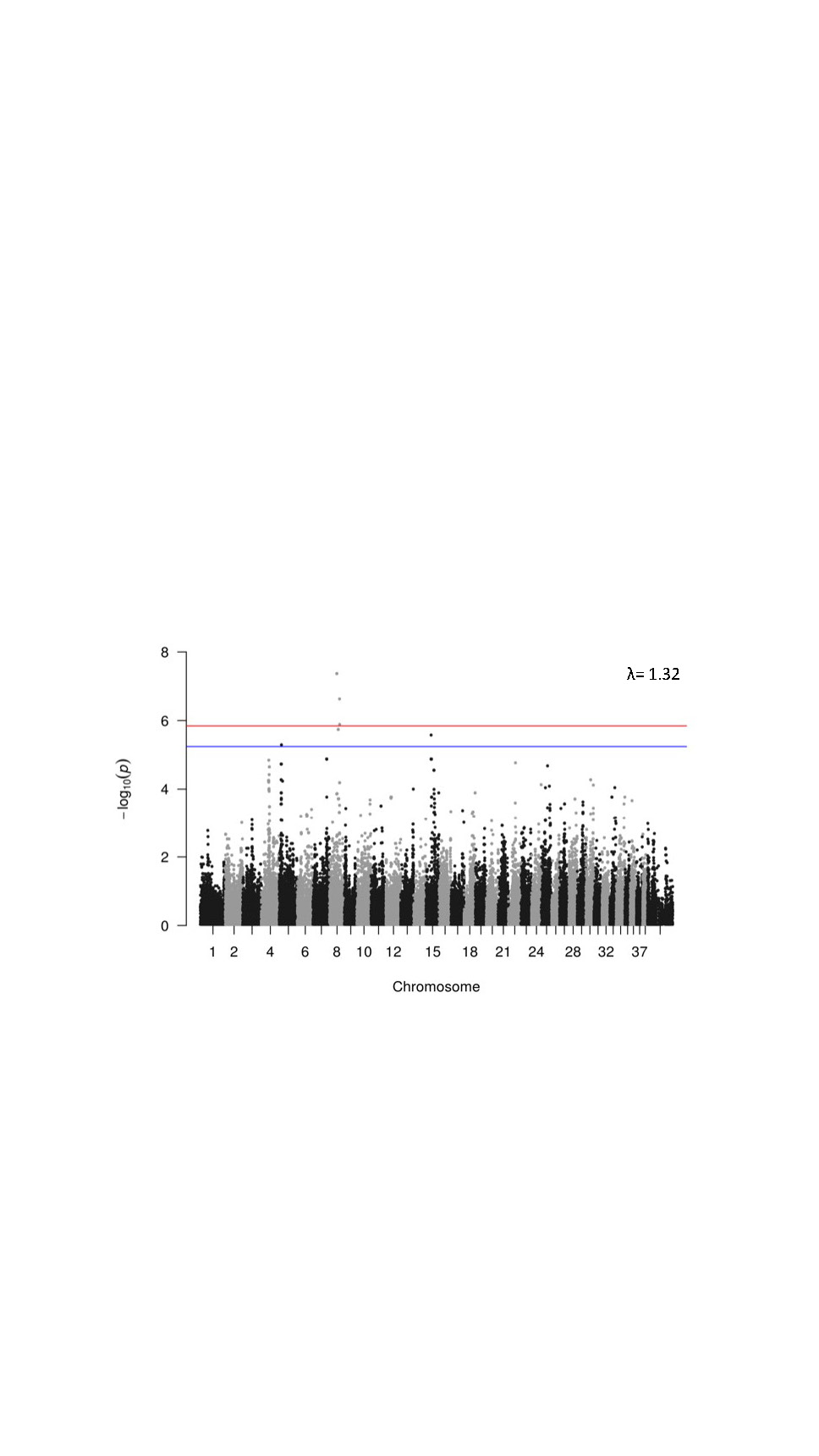


Supplemental File 2 Table 1. Odds ratios of the significantly associated SNPs on CFA 8 in a small subset of BT (n = 5) whose IE was fully responsive to phenobarbital compared to BT controls (n = 47). The CanFam3.1 reference allele is indicated in bold and the allele associated with elevated risk for IE is indicated with an asterisk.

| **SNP** | **Location (bp)** | **Allele** | **Controls (2n=94)** | **Cases (2n=10)** | **OR (95% CI)** | **p value** |
| --- | --- | --- | --- | --- | --- | --- |
| BICF2P1028102 | 35,194,728 | A* | 2 | 4 | 30.67 (4.64-202.52) | 0.000621 |
|  |  | **G** | 92 | 6 |  |  |
| BICF2P795913 | 36,051,327 | C* | 2 | 4 | 30.67 (4.64-202.52) | 0.000621 |
|  |  | **T** | 92 | 6 |  |  |
| BICF2P601314 | 49,002,573 | A* | 6 | 5 | 14.67 (3.31-65.09) | 0.000973 |
|  |  | **C** | 88 | 5 |  |  |
| BICF2P623511^ | 49,484,158 | G | 89 | 6 | 0.051 (0.01-0.28) | 0.001486 |
|  |  | **T*** | 3 | 4 |  |  |

^ genotype was missing for one control dog
